# Avoiding Under- and Overrecruitment in Behavioral Intervention Trials Using Bayesian Sequential Designs: Tutorial

**DOI:** 10.2196/40730

**Published:** 2022-12-16

**Authors:** Marcus Bendtsen

**Affiliations:** 1 Department of Health, Medicine and Caring Sciences Linköping University Linköping Sweden

**Keywords:** digital alcohol intervention, Bayesian sequential design, sample size, randomized controlled trial, trial recruitment, behavioural intervention, participant recruitment, research participants, research methods, effect size, trial procedure

## Abstract

Reducing research waste and protecting research participants from unnecessary harm should be top priorities for researchers studying interventions. However, the traditional use of fixed sample sizes exposes trials to risks of under- and overrecruitment by requiring that effect sizes be determined a priori. One mitigating approach is to adopt a Bayesian sequential design, which enables evaluation of the available evidence continuously over the trial period to decide when to stop recruitment. Target criteria are defined, which encode researchers’ intentions for what is considered findings of interest, and the trial is stopped once the scientific question is sufficiently addressed. In this tutorial, we revisit a trial of a digital alcohol intervention that used a fixed sample size of 2129 participants. We show that had a Bayesian sequential design been used, the trial could have ended after collecting data from approximately 300 participants. This would have meant exposing far fewer individuals to trial procedures, including being allocated to the waiting list control condition, and the evidence from the trial could have been made public sooner.

## Introduction

### Overview

Substantial effort is often expended on recruiting and collecting data from participants in behavioral intervention trials. Delivering interventions to participants often incur additional costs that need to be considered in restricted budgets. These efforts and costs need to be balanced with study objectives, as increasing the number of participants leads to reduced uncertainty in effect estimates. It is, therefore, not surprising that sample size considerations are given serious attention during the planning of trials, mixed in with feelings of despair, disbelief, and above all, hope.

With misguided faith in null hypothesis testing delivering certainty about effects in otherwise uncertain circumstances [1], power calculations to determine sample sizes have become a staple in study protocols as well as in ethics approval and grant applications. Fixating the risks of false negatives and false positives (power and significance) at widely adopted rates, researchers conducting power calculations tend to focus on the magnitude of effects they wish to detect as the variable dictating sample sizes. However, effects of interventions are uncertain, which is precisely why trials are conducted in the first place, and so deciding on the magnitude of effect a priori is in practice impossible. What sometimes then happens is that researchers, in fear of underrecruiting and not having enough power to detect statistically significant effects, pick the smallest effect size that they would not want to miss [2,3]. This results in unnecessary costs and efforts to recruit, intervene, and collect data from participants if effect sizes turn out to be greater than this minimal effect size. Other times, the effect size is assumed to be unreasonably large to reduce the required sample size and convince ethics and grant boards that the trial is feasible [2]. This leads to underrecruiting, and it leads to the null hypothesis not being rejected, and as is often the case, misinterpreted to be evidence of no effect, despite the existence of an observable difference between groups [4,5].

Over- and underrecruiting participants is both costly and unethical [3]. It leads to subjecting more than necessary participants to unnecessary effects from study procedures [6], potentially harmful or noneffective interventions and control conditions [7-9], or ending a trial with ambiguous findings when recruiting more participants could deliver less uncertain evidence [10]. One solution is to abandon a priori fixed sample sizes altogether, letting the data collected during the trial dictate when recruitment should end. Bayesian sequential designs are examples of this approach [11-13], where data are continuously analyzed and decisions are made throughout the trial period on whether or not recruitment should end.

### Objective

The objective of this study is to demonstrate how a recently completed trial of a digital alcohol intervention would have played out had a Bayesian sequential design been used, rather than following a traditional fixed sample size based on a priori power calculations. We will show that participants were excessively overrecruited, resulting in costs and efforts wasted when the evidence was already at hand.

## Bayesian Statistics and Sequential Designs

The literature on Bayesian statistics and sequential designs is substantial [1,10-13], and readers should have no problem finding in-depth descriptions. Therefore, we will introduce both, while at the same time assuring readers that they should feel comfortable moving on to the real-world examples even if not all details in this section are understood.

### Bayesian Statistics

**Figure 1:**
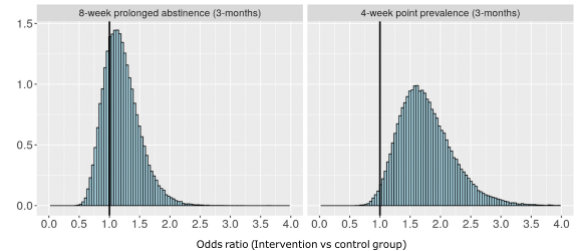
Marginal posterior distributions of odds ratios for smoking cessation (prolonged abstinence and point prevalence of smoking abstinence)—comparing study participants who had access to a digital smoking cessation intervention versus waiting list control group participants.

To understand Bayesian sequential designs, one needs to have at least a general understanding of Bayesian statistics. Within the Bayesian paradigm, one is interested in estimating the *posterior probability distribution* of parameters. In trials, the parameter that is given the most attention is the one that represents the effect of the intervention. The posterior probability distribution tells us how likely different parameter estimates are relative to one another. For instance, in a trial of a smoking cessation intervention, we could report the probability that the odds ratio (OR) of successful smoking cessation is greater than 1, that it is greater than 1.5, or that it lies between 0.9 and 1.1, and so on. As a concrete example, Figure 1 show two posterior probability distributions over OR estimated from a trial of a digital smoking cessation intervention among high school students [14-16]. The posterior distributions in Figure 1 show us that the effect of the intervention on 8-week prolonged abstinence from cigarettes 3 months post baseline (left plot) was approximately 1.2, and that 73.8% of the posterior probability distribution was above an OR of 1—leaving some uncertainty about the effectiveness of the intervention on this outcome measure. The right plot in Figure 1 shows that the OR for 4-week point prevalence of abstinence from smoking was approximately 1.8, and that 98.4% of the posterior probability distribution was above an OR of 1, suggesting strong evidence that there was a difference between groups with respect to this outcome measure.

The posterior probability distribution is calculated by combining the information available through the data collected, with what is known as the *prior probability distribution*—known simply as the *prior*. The prior represents our belief regarding the parameters before we collect data (ie, in a trial, the prior represents our belief about the effects before the trial commenced). The prior can be used to take a skeptical stance regarding effects by centering the prior around the null or to incorporate findings from previous studies by centering the prior around effect sizes estimated in previous trials. When data are scarce, the prior will influence the posterior distribution to a larger extent but will fade away as more data are collected. When using skeptical priors, this means that effect estimates are pulled toward the null when data are scarce, which is a powerful method of ensuring that conclusions of effects are not drawn prematurely using small sample sizes.

**Figure 2:**
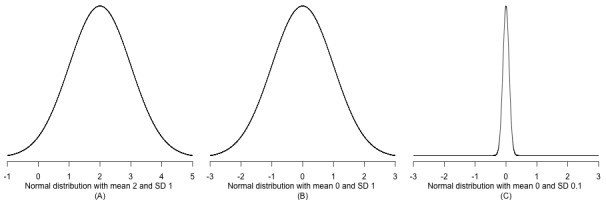
Examples of prior distributions; (A) normal distribution with mean 2 and SD 1; (B) normal distribution with mean 0 and SD 1; (C) normal distribution with mean 0 and SD 0.1.

To illustrate this, Figure 2 shows three prior distributions. In Figure 2A, a prior distribution in the form of a normal distribution with a mean of 2 (SD 1) is shown. This prior says that, before we see any data, we believe that the effect of the intervention being studied is most likely to be around 2 but we also are uncertain about this, encoded by the width of the distribution. In Figure 2B, a prior distribution in the form of a normal distribution with a mean of 0 (SD 1) is shown. This prior says that, before we see any data, we believe that the effect of the intervention is most likely to be around 0 (ie, taking a skeptical stance); similarly, we are encoding uncertainty in this assumption through the width of the distribution. Finally, in Figure 2C, a prior distribution in the form of a normal distribution is shown, which is centered at 0 but has a SD of 0.1 and is therefore much narrower than the normal distributions in Figure 2A and Figure 2B. This prior encodes a strong belief that the effect is close to 0. In all cases, the priors influence on the final posterior probability distribution will be strongest when there are fewer data points; thus, when the sample size grows, the data speak louder than the prior, and our prior beliefs will be overridden by the data.

### Bayesian Sequential Designs

Rather than targeting a fixed sample size, a trial adopting a Bayesian sequential design aims to recruit enough participants so that the posterior distribution of the effect estimate is informative relative to the study objectives. For instance, in a trial of a smoking cessation intervention, where our main concern is the OR of abstinence, we may decide that we want to show that the posterior probability of the OR being greater than 1 is at least 89% (or any other probability we find sufficient relative to the study context). Therefore, we collect data and continuously analyze it until we have reduced the uncertainty enough so that we can show that the OR is greater than 1 with at least 89% probability. There is, however, no need to have only one target; rather, it is often reasonable to include at least one more target defining when the intervention seems ineffective and it is futile to continue the trial. An example of this would be if the posterior probability is at least 92% that the OR is greater than 0.9 and less than 1.1 (ie, close to the null). The targets, often referred to as *criteria*, are succinctly expressed using formal notation. Thus, for the smoking cessation intervention trial example given above, the target criteria could be as follows:

- Effect: p ( OR > 1 | D ) > 89%
- Futility: p ( 0.9 < OR < 1.1 | D ) > 92%
- Harm: p ( OR < 1 | D ) > 89%

Note that criteria should be defined relative to the study objectives, the context in which they are evaluated, and their potential benefits and harms. If one was evaluating the effects of a surgical procedure, perhaps the 89% probability of effect should be closer to 98% probability, while the probability for harm should perhaps be revised down to 75%.

## A Trial of a Digital Alcohol Intervention

### Overview

To demonstrate how a trial may develop using a Bayesian sequential design in contrast to a fixed sample size, we revisit a randomized trial of a digital alcohol intervention [17,18]. The effects of the intervention were estimated using a 2-arm parallel group trial, where one group was given access to the intervention for 4 months, while the other group was given information about alcohol and health aimed to motivate them to drink less and given access to the intervention after the trial. The trial was prospectively registered in the ISRCTN registry (48317451).

### Ethics Approval

The trial received ethics approval on November 6, 2018, by the regional ethical committee in Linköping, Sweden (DNR 2018/417-31).

### Study Procedures

In this tutorial, we will only give a brief overview of the trial procedures; a full description of the trial is available in the study protocol [18]. The target population was Swedish-speaking adults seeking help on the internet to reduce their alcohol consumption. Individuals were required to be at least 18 years of age, have access to a mobile phone, and be classified as risky drinkers according to Swedish guidelines. Participants who showed interest in the study and gave informed consent were asked to respond to a baseline questionnaire (which also assessed eligibility) and were subsequently randomized. Participants were not blind after allocation, as they were aware whether or not they received immediate access to the digital intervention.

The core element of the digital intervention was a text message sent to participants each Sunday afternoon. The text message included a prompt to self-monitor one’s current alcohol consumption, with a hyperlink to a web-based tool. Those who decided to click on the link were asked to report their recent drinking and were then given access to personalized support. More information on the intervention is available in the study protocol [18].

Participants allocated to the control group were advised that they would receive information designed to motivate them to think more about reducing their alcohol consumption and that after 4 months they would receive additional support delivered to their mobile phone. Participants in the control group also received a single text message with basic health information regarding short- and long-term effects of alcohol consumption that also included a link to a website with information about alcohol.

### Outcomes and Follow-up

There were two primary outcomes in the trial, as follows:

- Frequency of heavy episodic drinking (HED), which was assessed by asking participants how many times they consumed 4 (for women), 5 (for men), or more standard drinks on one occasion the past month.
- Total weekly alcohol consumption (TWC), which was measured using a short-term recall method by asking participants the number of standard drinks consumed the past week.

Outcomes were assessed at 2- and 4-month postrandomization, initiated by sending text messages to participants with hyperlinks to questionnaires. Participants were called to collect responses if there was no response to reminders.

### Original Sample Size Calculation

The required sample size was determined using Monte Carlo simulations. A full description of the simulations is available in the study protocol [18]; thus, for succinctness, we restrict the description in this tutorial to the most relevant parts. We believed that a minimal relevant effect for the type of intervention studied, taking into consideration the unguided nature of the intervention and the setting, would be if the intervention group were consuming 15% less alcohol per week at the 4-month follow-up in comparison to the control group. We aimed for an expected power of 80% at the 0.05 significance threshold. Based on our previous studies of digital interventions in Sweden, we expected an attrition rate between 5% and 25%. The simulations suggested an expected sample size of 2126 individuals (interquartile range 2031-2198).

Participants were recruited over a series of 6-month periods. Between each period, we checked if the planned sample size had been achieved. Between April 25, 2019, and November 26, 2020, at which time recruitment was stopped, we randomized 2129 participants. This equated to approximately 19 months of recruitment, having allowed an initial grace period of 1 month for advert placement algorithms to optimize their performance.

### Estimates Over Time

**Figure 3:**
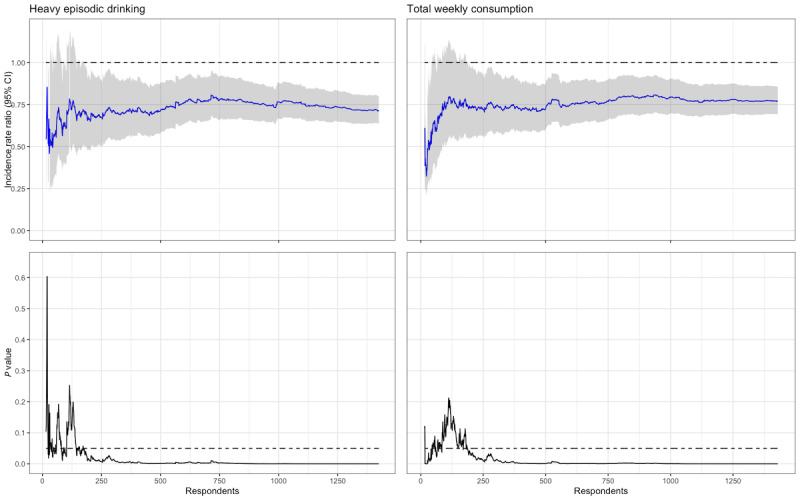
Maximum likelihood estimates and *P* values plotted against the number of respondents.

Putting aside the required sample size of 2129 participants, what would our null hypothesis-based analyses have looked like if we had stopped the trial after collecting data from only 15 participants? What about after 100 or 200 participants? In Figure 3, two pairs of plots are presented that show our analyses of HED and TWC given a certain number of responders to the 4-month follow-up. Looking at Figure 3, we can see in the plots on the top row the incidence rate ratio (IRR) and 95% CI. The analyses showed an IRR less than 1 (ie, the intervention group was drinking less than the control group) already from the first few responders. In the bottom row, the *P* value can be seen to fluctuate heavily in the beginning, crossing the line of statistical significance on multiple occasions and settling below the .05 line at approximately 200 responders. After 200 responders, the IRR estimates (top row) continue to move around somewhat but staying close to approximately an IRR of 0.75. In our main analyses of the trial [17], which included the full sample size, we concluded that the IRR for TWC was 0.77 (95% compatibility interval 0.69-0.86), and the IRR for HED was 0.71 (95% compatibility interval 0.63-0.79)—findings that were already at hand if we had stopped recruiting after collecting data from approximately 250 participants (ie, 12% of the planned sample size).

### Bayesian Sequential Design

If we had decided to not use a fixed sample size but had rather adopted a Bayesian sequential design, we would have foregone a power calculation and instead defined target criteria for when recruitment should end. These criteria may have been the following:

- Effectiveness: p ( IRR < 1 | D ) > 97.5% and p ( IRR < 0.87 | D ) > 50%
- Futility: p ( 0.87 < IRR < 1.15 | D) > 97.5%

The effectiveness criterion says that we should stop recruitment if the probability that the intervention group is drinking less than the control group is greater than 97.5%; it also says that the probability of the estimated IRR being less than 0.87 should be greater than 50%. An IRR of 0.87 is comparable with our fixed sample size power calculation assumption of 15% less alcohol consumption in the intervention group versus the control group. The futility target criterion says that we will stop recruitment if it is more than 97.5% likely that the estimated IRR is between 0.87 and 1.15, that is, within a range of effect sizes that are considered too small to be of importance considering the context.

Just like we did for the null hypothesis analyses in Figure 3, we can plot the target criteria over time to see what they would look like given a certain number of participants. Since these are Bayesian analyses, we must decide on priors for coefficients before we do inference. In our demonstration, we compare the use of standard normal priors, that is, normal distributions with a mean of 0 (SD 1), with more conservative normal priors, with a mean of 0 (SD 0.1), as in Figure 2B and Figure 2C.

**Figure 4:**
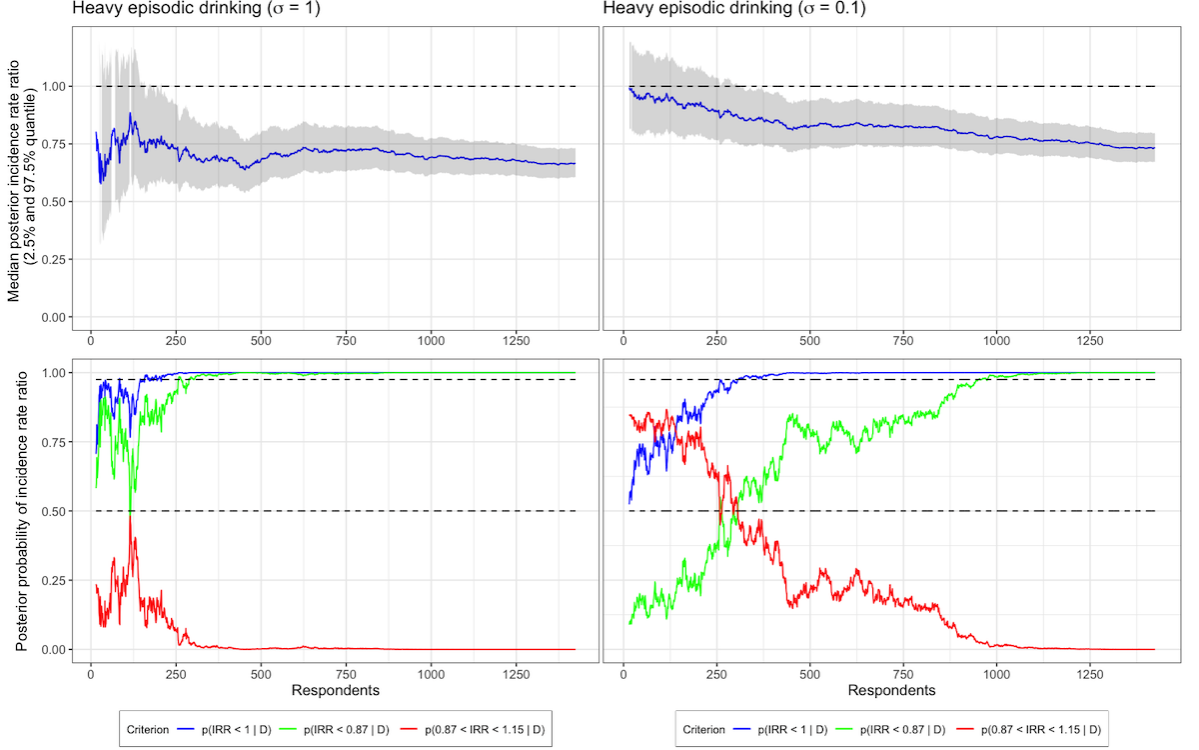
Posterior probability distributions and target criteria plotted over available data from respondents with respect to total weekly consumption (TWC) using both standard normal priors (left) and skeptical priors (right). IRR: incidence rate ratio.

Figure 4 shows, for HED, the evaluated target criteria over number of respondents. In the top left plot, we see the median of the posterior distribution of the IRR using standard normal priors (ie, SD of 1). The analysis shows that the estimated effect was in the direction of the intervention group consuming less alcohol than the control group already early in the trial. In the bottom left plot of Figure 4, the effectiveness criteria are represented by the blue and green lines and the futility criteria by the red line. As it can be seen, after approximately 225 participants, the criteria are fulfilled, and the trial could have ended with evidence of the intervention producing lower HED. However, scrutiny of the bottom left plot shows that the same conclusion could have been made after 175 participants, as the criteria were fulfilled briefly. Generally, we would like to avoid making conclusions with small sample sizes, and the plots show that findings are not stable early on. We can avoid making claims when data are scarce by encoding skepticism using priors. In the two plots on the right in Figure 4, the same analyses are presented but with a normal prior distribution with a SD of 0.1. As it can be seen in the top right plot, effect estimates are strongly pulled toward an IRR=1 at the beginning of the trial, and in the bottom right plot, both effect criteria are below their respective target lines. It is not until after approximately 300 participants that the criteria settle down and show strong evidence that the intervention has a positive effect on alcohol consumption. This *shrinkage* of estimates plays a crucial role in protecting from spurious findings when data are scarce.

**Figure 5:**
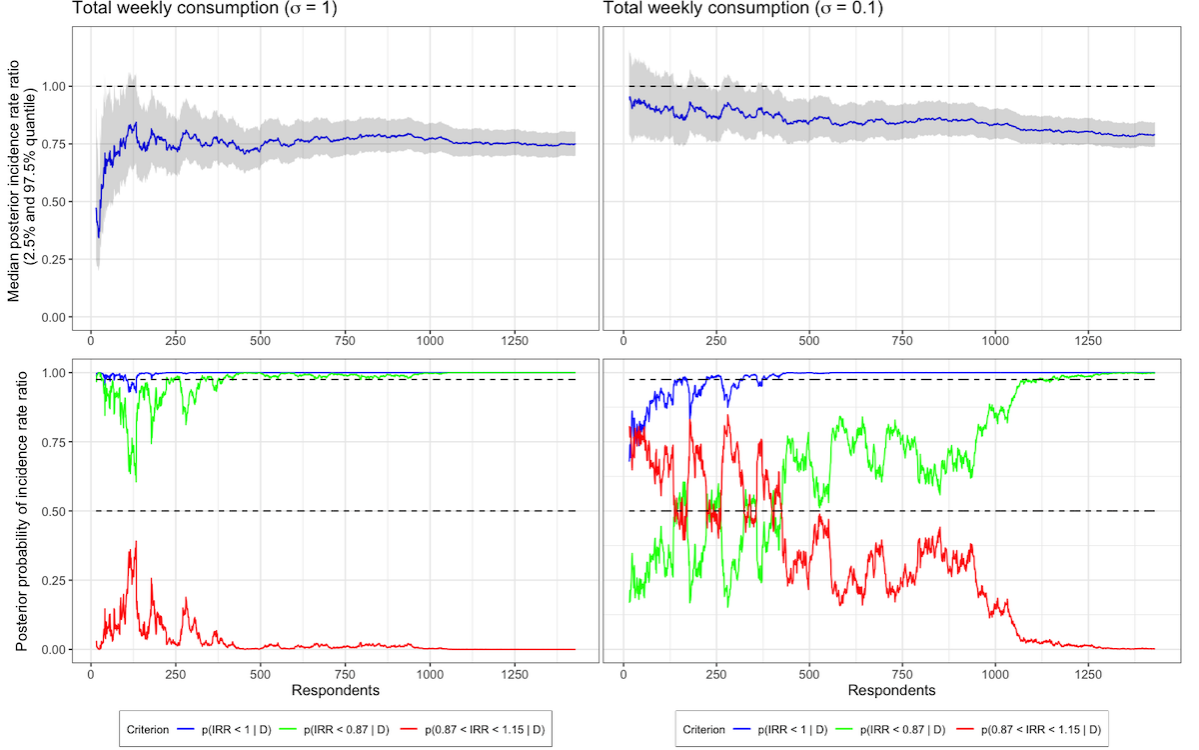
Posterior probability distributions and target criteria plotted over available data from respondents with respect to total weekly consumption (TWC) using both standard normal priors (left) and skeptical priors (right). IRR: incidence rate ratio.

In Figure 5, IRR estimates and effect criteria are plotted for TWC. In the left plots, we can see that the effectiveness criteria are above their respective lines (97.5% and 50%) already after 15-20 participants, and then they begin to waver until settling down after approximately 300 participants. The futility criterion never comes close to crossing the 97.5% line. Although mathematically correct, most researchers should feel uneasy about making claims of effectiveness after only 15-20 participants, and the left-hand side of Figure 4 shows rightly that. Again, skepticism encoded in the prior may help, and as it can be seen in the plots on the right in Figure 5, early claims of effect are protected against. However, scrutiny of the right-hand side of Figure 5 also reveal that there are multiple instances when both effectiveness criteria are fulfilled but later cross below their target lines again, prior to data being available from 300 participants. Even more skeptical priors may protect against these early findings; however, good judgement from researchers when studying the development of their evidence may be more effective.

## Discussion

A trial of a digital alcohol intervention could have stopped recruitment after approximately 15% of the prespecified sample size had been recruited if a Bayesian sequential design had been used. The consequences would have been fewer participants recruited to a control condition that made them wait for the novel support tool and reduced costs of recruitment; in addition, evidence of the intervention’s effectiveness could have been made public sooner. Instead, overrecruitment was the result of anticipating small effects from a public health intervention of this type, while also controlling for the risk of type 1 and 2 errors.

Trials are conducted because effects of interventions are not known; thus, the design of trials should facilitate discovery efficiently. This is not to say that prior knowledge cannot be useful when designing Bayesian sequential designs; on the contrary, both conservative views on the effects and data from previous trials can be incorporated into the priors used during analysis. Priors are ideal in this circumstance since they dominate the analysis when data are scarce, protecting from spurious findings, yet their influence is lessened as more data become available.

Bayesian sequential designs do not rely on an a priori fixed sample size; nevertheless, planning, ethics approval, and grant applications often require one. This can still be achieved by estimating the final sample sizes using simulation [12]. Statistical software can generate synthetic data simulating the planned trial, and analyses can be done using these synthetic data to evaluate the criteria specified in the trial design. By repeating this procedure multiple times, with varying effect sizes, an estimate of how many participants it will require to fulfill the criteria can be produced.

One caveat that should be avoided when using Bayesian sequential designs is to view the target criteria as hard and fast rules—making them shortcuts to going back to dichotomizing evidence into effect and no effect. Instead, the target criteria should be viewed as researchers’ intentions for what is considered findings of interest. One may have fulfilled some criteria of the trial but not others and still decide to end the trial. The trial should be stopped when, on the basis of accumulated results, the answer to a scientific question is sufficiently well known that the results can be used in a broader context [12]. The posterior distribution of effect can be estimated and reported, with the probability of a difference between groups indicating the certainty about findings.

In some trials, it will not be possible to access follow-up data continuously throughout the trial period to check the criteria, and so a Bayesian sequential design may not be possible to adopt. This may be the case if data are collected at multiple sites, possibly internationally, and it is time-consuming to collate all data to do analyses. However, it should be noted that the benefits of sequential designs may still be used in cases where it is possible to analyze data at least occasionally, for instance for every 50-100 participants. Analyses do not have to be done for every new data point available but rather for larger sets of participants.

Finally, reducing research waste and protecting research participants from unnecessary harm should be top priorities for researchers studying interventions. To avoid under- and overrecruitment, which occurs when using fixed sample sizes, is an important mitigation, and Bayesian sequential designs allow for exactly this. Examples of their use in behavioral intervention trials can be found in the literature [19-22], and when appropriate, they should become standard procedure.

## Abbreviations

HED: heavy episodic drinking
IRR: incidence rate ratio
OR: odds ratio
TWC: total weekly consumption

## Data Availability

Deidentified data sets generated during or analyzed during this study will be made available upon reasonable request to the corresponding author, after approval of a proposal and with a signed data access agreement.
